# Structural changes of a bacteriophage upon DNA packaging and maturation

**DOI:** 10.1007/s13238-020-00715-9

**Published:** 2020-04-07

**Authors:** Wenyuan Chen, Hao Xiao, Xurong Wang, Shuanglin Song, Zhen Han, Xiaowu Li, Fan Yang, Li Wang, Jingdong Song, Hongrong Liu, Lingpeng Cheng

**Affiliations:** 1 Key Laboratory for Matter Microstructure and Function of Hunan Province, Key Laboratory of Low-dimensional Quantum Structures and Quantum Control, School of Physics and Electronics, Hunan Normal University, Changsha, 410081 China; 2 State Key Laboratory for Infectious Disease Prevention and Control, National Institute for Viral Disease Control and Prevention, Chinese Center for Disease Control and Prevention, Beijing, 100052 China; 3 Technology Center for Protein Sciences, School of Life Sciences, Tsinghua University, Beijing, 100084 China; 4 State Key Laboratory of Microbial Resources, Institute of Microbiology, Chinese Academy of Sciences, Beijing, 100101 China

**Dear Editor,**

Tailed, double-stranded DNA (dsDNA) bacteriophages, which belong to the order of Caudovirales, have a tail attached to a pentameric vertex of the icosahedral capsid shell (head) through a 12-fold portal (Johnson and Chiu, 2007). The phages package genomic dsDNA into a round procapsid using the portal in complex with an ATP-dependent terminase complex as the motor. During packaging, the procapsid shell expands to a more angular intermediate to match the size of the viral genome (Guo et al., 2014). When the phage head is full, the portal detects internal pressure and conveys a signal from the inner capsid to the exterior, which triggers a sequence of events—the terminase complex cleaving mature DNA genome from a multi-genome concatemer, the release of the terminase complex from the portal, and the attachment of the tail complex—in the completion of phage assembly (Lander et al., 2006; Johnson and Chiu, 2007). At the beginning of phage infection, the tail is responsible for receptor recognition, and the portal and tail act as a tunnel for DNA delivery into the host cytoplasm (Johnson and Chiu, 2007). These mechanisms of DNA packaging and ejection may also be conserved in many other DNA viruses, including herpesvirus (Wang et al., 2020; Yang et al., 2020).

In previous studies, the structures of whole phage capsid, isolated portal, and tail components have been determined at medium to near-atomic resolutions (Fokine and Rossmann, 2014; Prevelige and Cortines, 2018). Such structural information indicates that tailed phages use the conserved structures of the capsid shell protein, as well as the DNA packaging and ejection machinery, implying strong functional similarities. However, the high-resolution *in situ* structures of the portal and tail are still less well understood, probably due to the intrinsic flexible assembly of these components and the overlap of different symmetrical components in the phages. The molecular mechanism through which events are detected by the portal and subsequently signaled to the exterior terminase complex to complete the phage assembly remains unclear.

*Escherichia coli* bacteriophage T7, a member of Podoviridae, has been used as a model for understanding the DNA packaging mechanism common to tailed phages and related dsDNA viruses (Guo et al., 2013; Hu et al., 2013; Guo et al., 2014; Cuervo et al., 2019). Here, we determined structures of the DNA packaging intermediate and mature capsids of T7 using cryo-electron microscopy (cryo-EM) and symmetry-mismatch reconstruction. The structural resolutions of the portal, portal-tail complex, and cores (ejection proteins) in the two capsids have been further improved to 3.8–6.0 Å through a symmetry-mismatch and local reconstruction method that we developed. Our portal and tail structures have different conformations than recently reported recombinant portal and tail structures of T7 (Cuervo et al., 2019). The portal interacts flexibly with the capsid shell and core in both mature and intermediate capsids. Our structures reveal the conformational changes of the portal, core, and shell from the DNA packaging state to the mature state, and provide insights into the head-full packaging mechanism of the phages.

The mature T7 phage was purified from cells for the cryo-EM structural analysis (Fig. 1A). We obtained the mature phage icosahedral capsid shell structure at 3.5 Å resolution by using the cryo-EM and icosahedral reconstruction method (Figs. S1A and S2A–D). The icosahedral capsid shell structure is essentially identical to the previously published mature phage capsid shell structure (Guo et al., 2014). Subsequently, a 7 Å resolution asymmetric reconstruction of the mature phage with a portal-tail complex at one of the 12 icosahedral vertices (Fig. 1B) was obtained using the symmetry-mismatch reconstruction method (Liu and Cheng, 2015). The portal-tail complex structure is similar to the previously reported recombinant T7 portal-tail complex structure (Cuervo et al., 2019). The core structure is smeared in the asymmetric reconstruction. The portal and core are surrounded by DNA within the capsid, and a bulk of disordered condensed DNA is stacked on the top of the core (Fig. 1B). Such DNA organization is commonly observed in other tailed phages (Johnson and Chiu, 2007).

**Figure 1:**
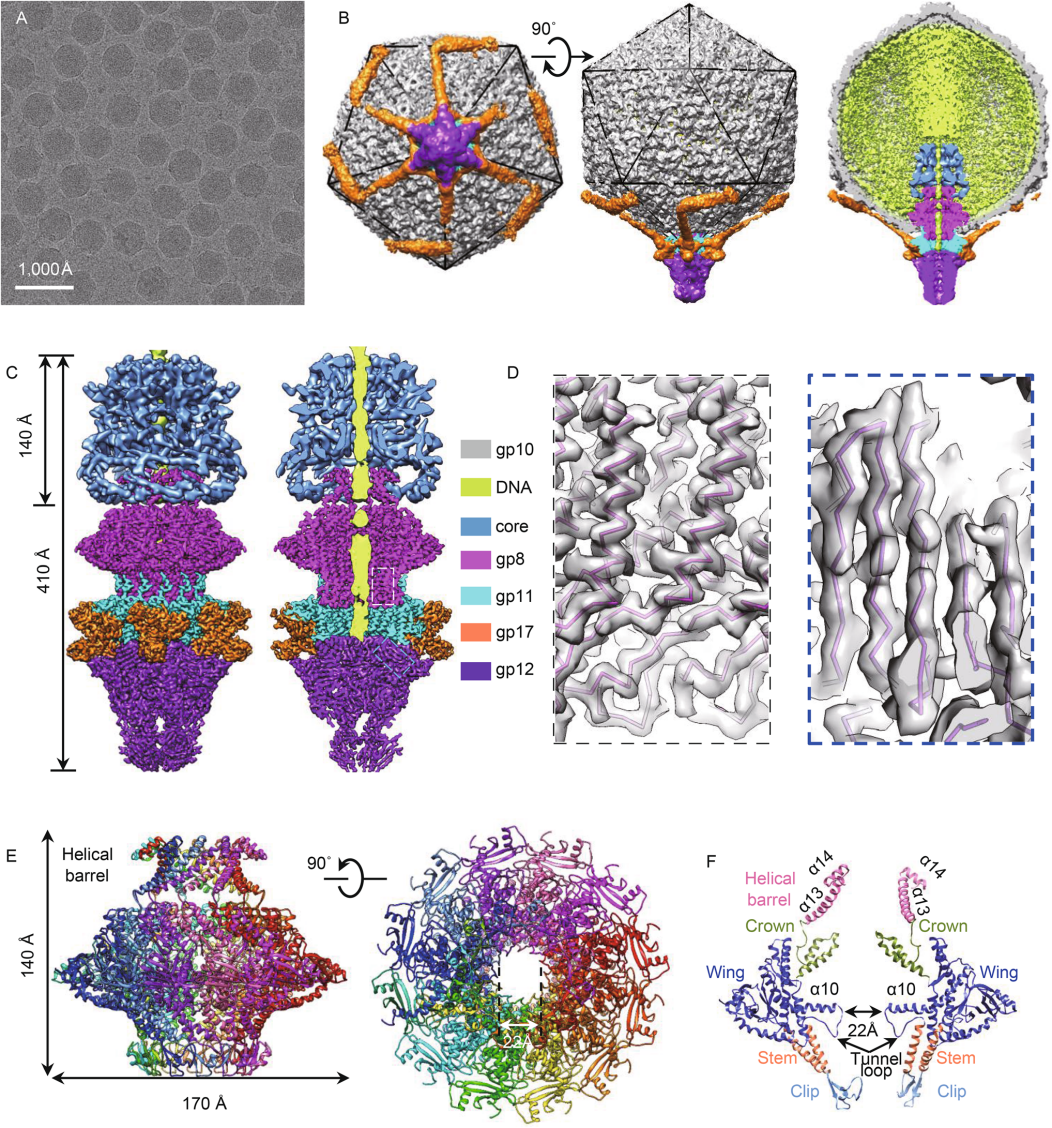
**Structure of the mature T7 phage**. (A) Cryo-EM image of mature phage T7. (B) Surface and cut-open views of the asymmetric structures of mature T7 phage. (C) Surface and cut-open views of the core (blue), portal (magenta), adaptor (cyan), fiber N-terminus (orange), and nozzle (purple). The regions of portal-tail complex and core were reconstructed using independent local reconstructions (D) Zoom-in views of the boxed regions in the panel C. The transparent density maps are superposed with the atomic models (only backbones are shown). (E) Side and top views of the atomic model of portal in the mature T7. (F) Slab view of the portal. Only two copies of gp8 are shown. The portal domains are in different colors

We performed the local refinement and reconstruction focusing on the region of the portal, tail, and core. The portal-tail complex was reconstructed at the same resolution of 3.8 Å (Figs. 1C, 1D and S1B), although the core structure was still smeared. Independent local reconstruction focused on the core yield the structure of the core at the resolution of 6.0 Å (Figs. S1B and S3). Another two independent local reconstructions focused on the portal and tail, respectively, yield the structures of the portal and tail at the same resolution of 3.8 Å, suggesting that the portal interacts firmly with the tail. The improved resolutions of the portal-tail complex and core structures by using the independent local reconstructions suggest that the portal interacts flexibly with the core and the capsid shell. Our analysis of the orientation distributions of the portal with respect to the shell in the mature particles suggests that many of the particles have a varied ϕ angle around the portal axis assigned to the portal, requiring a change of up to approximately 3° or −3° (a span of 6°) in either direction during local refinement (Supplementary Materials). It is intriguing that considering that the interaction of the portal and the shell is a symmetry mismatch between 12- and 5-fold, if the portal could turn 6° axially, then the portal-shell interaction would coincide with the portal-shell interaction before the turn (Fig. S4). The lower resolution of the core structure can be ascribed to its central location, in which the DNA affects the local refinement. The whole core was resolved as an 8-fold symmetrical structure (Figs. 1C and S3) similar to the core structure in the T7 procapsid (Guo et al., 2013). The bottom region is a helix-rich structure.

The T7 portal, which exhibits a canonical 12-fold phage portal structure, is formed by 12 copies of the 536-residue portal protein gp8 arranged around a central tunnel (Figs. 1E and S5A). Each gp8 can be divided into five domains of clip (294–334), stem (335–356 and 269–293), wing (1–268, 357–427), crown (428–492), and C-terminal helical barrel (Fig. 1F). The tunnel is 140 Å in height and of variable tunnel diameter. The narrowest channel is approximately 22 Å in diameter and is formed by a so-called tunnel loop (Lebedev et al., 2007) and a long kinked α-helix (α10) in the wing (Fig. 1F). The 40-Å long C-terminal helical barrel is formed by 12 right-handed twisted α-helices (α13), in stark contrast to the 200-Å long left-handed twisted phage P22 portal helical barrel (Olia et al., 2011). An extra rim of the top opening, which is formed by 12 C-terminal α-helices (α14) and loops, interacts with the core (Fig. 1F).

Twelve copies of adaptor protein gp11 assemble around the bottom of the portal forming a ring-like structure and serving as the base for the assembly of a hexameric tube consisting of six copies of nozzle protein gp12 (Fig. S6A–C). Six trimers of the tail fiber gp17 N-termini anchoring to the adaptor-portal interface were clearly resolved (Figs. 1C, S6D and S6E). Each gp17 N-terminus is formed by a jelly-roll barrel and a 22-residue α-helix (Fig. S6D and S6E). The distal parts of the fibers, which are too flexible to be well resolved, were observed to radically extend back on the capsid shell at lower resolution (Fig. 1B).

A rod-like density that is approximately 20 Å in diameter and lining the middle of the portal and core channel could be assigned to dsDNA (Fig. 1B and 1C); this is similar to the rod-like density observed in the P22 portal (Lander et al., 2006). The dsDNA is embraced by the 12 tunnel loops in the portal, which have a weaker density probably due to its asymmetric interaction with the dsDNA groove. This dsDNA density terminates abruptly at the interface between the dodecameric ring of gp11 and hexameric nozzle of gp12 (Fig. 1B and 1C).

The DNA-free T7 capsid II particles, which were considered to be a DNA packaging intermediate subsequent to the T7 procapsid and morphogenetic precursor of the mature phage (Guo et al., 2014), were purified from cells for cryo-EM structural analysis (Fig. 2A). The icosahedral capsid II head structure is essentially identical to the mature phage (Fig. S2E and S2F) and is more angular compared with the round procapsid structure (Guo et al., 2014). No DNA molecule was observed to associate with the capsid II probably because the DNA molecule that the capsid II was packaging was detached during purification (Guo et al., 2014). The asymmetric reconstruction of the capsid II at about 10 Å resolution revealed a cylinder-shaped structure of the portal and core attached to the inner surface at one icosahedral vertex (Fig. 2B), in a similar organization to the previously reported T7 procapsid structures (Guo et al., 2013). The core structure on the top of the portal was also smeared (Fig. 2B).

**Figure 2:**
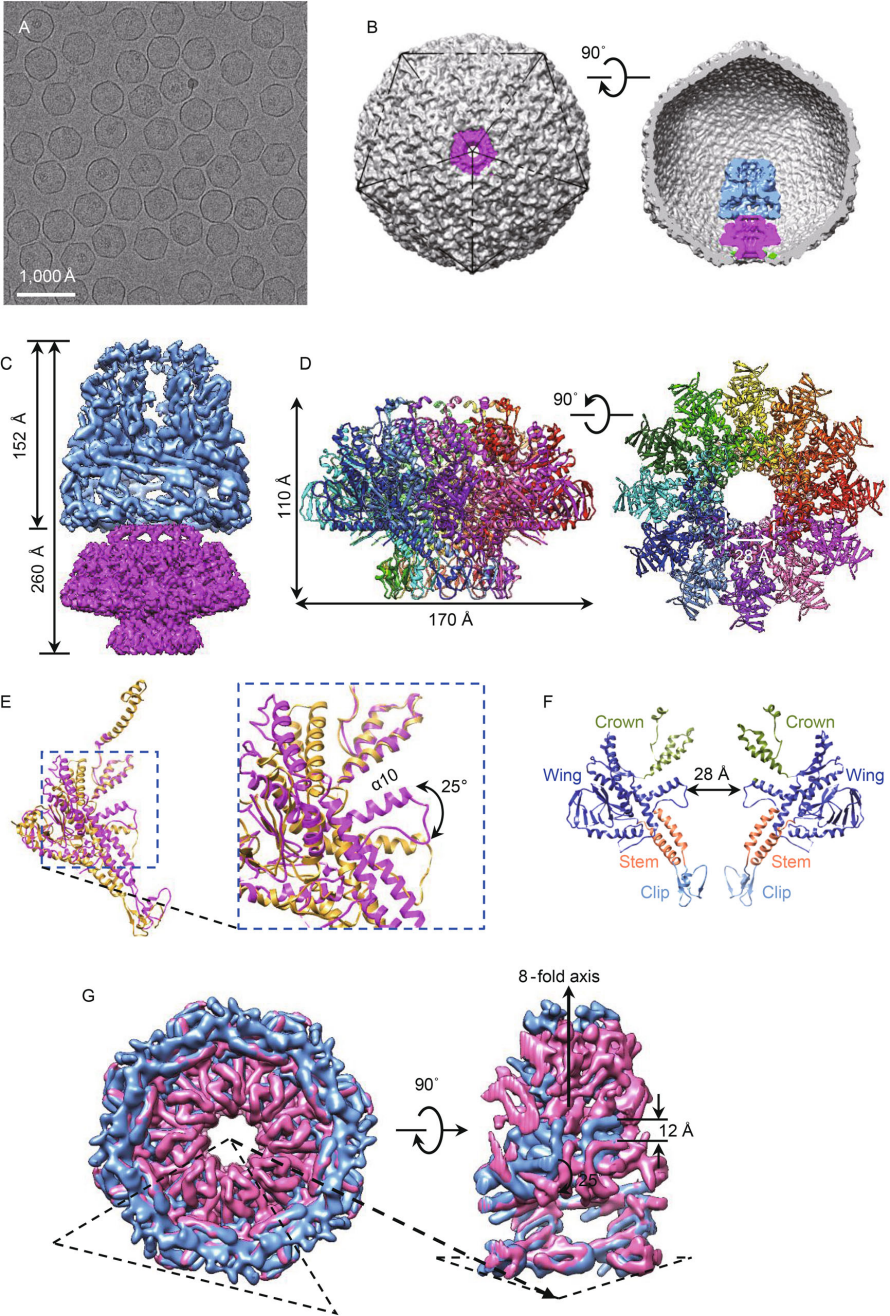
**Structure of T7 capsid II**. (A) Cryo-EM image of the capsid II particles. (B) Surface and cut-open views of the asymmetric structures of capsid II. (C) Structure of core (light blue) and portal (magenta) complex in capsid II. (D) Atomic model of the portal structure in capsid II. (E) Superposition of portal protein gp8 in capsid II (magenta) on the gp8 in the mature phage (yellow) shows that the wing undergoes a rigid-body rotation by approximately 25°. (F) Slab view of the portal. Only two copies of gp8 are shown. The color scheme of the portal domains is identical to that used in Fig. 1F. (G) Superposition of the core (pink) in the mature phage with the core (blue) in capsid II. A segmented view shows that the top region of the 8-fold core in the mature particle twists axially by approximately 25° and is compressed by 12 Å along the axis compared with that in capsid II

The local refinement and reconstruction improved the structural resolution of the portal to 4.9 Å (Figs. 2C, S1B, and S5B). The portal also interacts flexibly with the shell (Supplementary Material). The lower resolution of the portal structure in the capsid II suggests that the portal might be flexible to some extent or deviate from 12-fold symmetry during the DNA packaging. The portal in the capsid II is formed by 12 copies of symmetrically arranged protein gp8 with similar topology but different conformation (Fig. 2D and 2E) compared with the portal in the mature phage (discussed below). The absence of the barrel in our capsid II portal structure (Fig. 2D–F) and in the phage procapsid portal structures (Chen et al., 2011; Guo et al., 2013) suggests that the barrel is not involved in the DNA packaging. The portal clip region interacts with the rim of the open icosahedral vertex and five protrusions around the inner capsid surface (Fig. S7). The protrusion, which was resolved as two α-helices using the local reconstruction method, could not be assigned to the capsid shell protein gp10 (Fig. S7). The two α-helices, which is similar to the V-shaped α-helices structure in the P22 (Chen et al., 2011), could be assigned to the scaffolding protein interacting with the portal. This organization is consistent with a recent result that scaffolding protein interacts with portal subunits and catalyze the oligomerization of the portals (Yuan et al., 2018), implying a collaborative roles played by the portal, scaffolding protein, and shell protein in the capsid assembly. We did not resolve the structure of the terminase complex and DNA probably because they were lost during virus purification. We speculate that during DNA packaging the portal assumes the observed conformation. This conformation, however, may also reflect the DNA-free state of the purified capsid II.

The 8-fold core stacks on the crown and wing of the portal (Figs. 2B, 2C, and S8). The bottom region of the core is very similar to the core in the mature phage, but the top region undergoes a conformational change compared with the core in the mature phage (Fig. 2G, discussed below). According to the observation that the DNA is located along the core channel in the mature phage (Fig. 1B), the DNA may translocate along the core channel and the core may facilitate DNA organization during packaging.

A structural comparison between the portal in the capsid II and that in the mature phage revealed that the portal undergoes considerable conformational changes from the packaging intermediate to the mature state (Movie S1). The whole portal moves downward (Wang et al., 2020), resulting in the exposure of the clip (Fig. S9A). This movement causes a squeeze between the inner capsid shell and the wing. Therefore, the wing undergoes a rigid-body rotation by approximately 25° (Fig. 2E and Movie S1). Accordingly, the open vertex of the shell, which interacts with the portal, is squeezed out slightly by the portal (Fig. S9B). The clip tilts toward the portal axis, which probably triggers the disassociation of the terminase complex (Yang et al., 2020), followed by the assembly of the tail proteins. It is noteworthy that If the portal does not move downward and expose its clip domain out of the capsid shell, the tail is not able to assemble on the portal due to steric restriction. Accordingly, the tunnel loop channel becomes narrower (from 28 to 22 Å in diameter), and the stem channel becomes wider (from 25 to 40 Å in diameter). A structural comparison between the core in the mature phage and that in the capsid II indicates that the top region of the 8-fold core twists axially by 25° and the overall length is compressed by 12 Å along the axis in the mature phage (Fig. 2G). The axial length of the core becomes 140 Å in the mature phage (Fig. 1C), in contrast to the 152 Å in the capsid II (Fig. 2C).

Our in situ structural analysis of the portals in the mature phage and in the DNA packaging intermediate allows us to propose a model of the structural events involved in the transduction of the head-full signal and completion of the phage assembly. The head-full signal is first transduced from the packaged DNA to the core, and then to the wing and crown. When the phage head is about to be full, the lastly packaged DNA accumulates in the bulky region on the core (Fig. 1B) and pushes the portal outward via the core-portal interaction, resulting in the structural change in the portal. The structures of the twisted and compressed core (Fig. 2G) and squeezed open vertex of the shell (Fig. S9B) in the mature phage exactly demonstrate the DNA pressure. Concomitantly, the five copies of the scaffolding proteins around the 5-fold open in the shell (Fig. S7) finish their role of interacting with the portal clip, and are then expelled out of the shell. Consequently, the terminase complex cleaves the mature DNA genome from the concatemer and disassociates from the portal clip, followed by the association of the tail adaptor and nozzle to complete the phage assembly.

## FOOTNOTES

We thank the Computing and cryo-EM Platforms of Tsinghua University, Branch of the National Center for Protein Sciences (Beijing) for providing facilities and technical support. This research was supported by the National Research and Development Program of China (2016YFA0501103) and the National Natural Science Foundation of China (31971122 and 31570742), National Science Foundation of Hunan Province, China (2019JJ10002 and 2019JJ40096), and the Research Foundation of Education Bureau of Hunan province, China (19B372).

W.C., H.X., X.W., S.S., Z.H., X.L., F.Y., L.W., and J.S. performed experiments; J.S., H.L, and L.C. designed the study; all authors analyzed data; and H.L, and L.C. wrote the manuscript.

Wenyuan Chen, Hao Xiao, Xurong Wang, Shuanglin Song, Zhen Han, Xiaowu Li, Fan Yang, Li Wang, Jingdong Song, Hongrong Liu, and Lingpeng Cheng declare that they have no conflict of interest. This article does not contain any studies with human or animal subjects performed by any of the authors.

## Electronic supplementary material

Below is the link to the electronic supplementary material.

Supplementary material 1 (PDF 2713 kb)

Supplementary material 2 (MOV 11288 kb)
