# Supplementary material for: Structural changes of a bacteriophage upon DNA packaging and maturation
Source: Protein Cell. 2020 Apr 7;11(5):374–9. doi: 10.1007/s13238-020-00715-9 (PMC7196576; doi:10.1007/s13238-020-00715-9)
Supplement: Supplementary file 1 — Supplementary material 1 (PDF 2713 kb) [file 13238_2020_715_MOESM1_ESM.pdf]

SUPPLEMENTARY INFORMATION FOR:

Structural changes of a bacteriophage upon DNA packaging and maturation

Wenyuan Chen, Hao Xiao, Xurong Wang, Shuanglin Song, Zhen Han, Li Wang, Jingdong Song*, Hongrong Liu*, Lingpeng Cheng*

*Correspondence to: songjd@ivdc.chinacdc.cn, hrliu@hunnu.edu.cn, and lingpengcheng@mail.tsinghua.edu.cn

This PDF file includes:

Materials and methods
Supplementary text
Figures S1 to S11
Table S1
Legends for Movies S1
SI References

Other supplementary materials for this manuscript include the following:

Movies S1

Materials and Methods

Sample preparation.

T7 phages (ATCC, BAA-1025-B2) were inoculated in 1L *Escherichia coli* (ATCC, BAA102) cells for 4 h at 37℃. After the cells were lysed, phages in the supernatant were precipitated with 1M NaCl and 10% polyethylene glycol 8000 overnight. The precipitated phages were resuspended in the TNM buffer (50 mM Tris-HCl, 10 mM $MgCl_2$, 50 mM NaCl, pH 7.4) and then were purified on 1.56g/mL and 1.26g/mL CsCl cushions through ultracentrifugation. After centrifugation at 197,000 g for 12 h at 10℃, two virus bands were separated. The upper and lower band were collected and repeatedly ultracentrifuged on the aforementioned 1.26g/mL and 1.56g/mL CsCl cushions separately and dialyzed in TNM buffer overnight. Negative stain electron microscope observations indicated that the upper and the lower bands were empty and full particles, respectively.

Cryo-EM Imaging, image processing.

The full and empty particles of T7 were imaged with an FEI Technai Arctica 200 kV electron microscope equipped with a Falcon II camera at a nominal magnification of 78,000×, corresponding to a pixel size of 1.27 Å, respectively. The full electron dose of approximately 25 e^-/A^2 was fractionated into 30 movie frames, which were aligned and averaged to a single image (Li et al., 2013). The astigmatism and defocus value of each image were determined by a program that we designed. The viral particles were boxed automatically using the program ETHAN (Kivioja et al., 2000) and these boxes were then verified manually.

3D reconstructions of the unique vertices of the full and empty particles.

The icosahedral reconstructions of T7 full particles were performed using the programs (Li et al., 2017) based on the common-line algorithm (Fuller et al., 1996; Thuman-Commike and Chiu, 1997). To obtain the initial model of the asymmetric structure of the tailed phage, the location of the portal-tail complex for each particle image was determined based on the icosahedral orientation and center parameters through a search of the 12 icosahedral vertices for the unique vertex with the portal-tail complex. The correct orientation of the tailed phage for each particle image was determined using the symmetry-mismatch reconstruction method (Liu and Cheng, 2015). (i) For each particle image, we projected the model to generate 60 projection images according to the 60 equivalent icosahedral orientations of the particle image. We searched the 60 projection images for the projection image that best matches with the particle image and then assigned the corresponding orientation to the particle image. (ii) We reconstructed the tailed phage structure using the particle images according to the newly assigned orientations. We then obtained an improved model of the portal-tail complex. (iii) We iterated steps (i) and (ii) until the orientations of all particle images were mostly stabilized and the portal-tail complex structure could not be improved further.

We further refined the orientation for each particle image by using the symmetry-mismatch reconstruction method (Liu and Cheng, 2015). (i) We segmented the portal-tail complex region from the whole phage structure determined above for use as an initial model. (ii) For each particle image, we projected the 3D tail-portal complex model to generate 5 projection images according to the 5 equivalent orientations of the unique 5-fold capsid shell vertex. We searched the 5 projection images for the projection

image that best matches with the portal-tail region in the particle image and then assigned the corresponding orientation to the particle image, and this symmetry-mismatch search method is similar to that used for the bacteriophage ϕ29 reconstruction (Morais et al., 2001). (iii) We reconstructed a whole phage structure by using the particle images according to the newly assigned orientations. We then obtained an improved model of the portal-tail complex. (iv) We iterated steps (ii) and (iii) until the orientations of all particle images were mostly stabilized and the portal-tail complex structure could not be improved. A soft-mask was used for each particle image.

The orientation and center parameters of each portal-tail complex in the particle image were further refined using local refinement and reconstruction method (Wang et al., 2018; Yuan et al., 2018; Zhu et al., 2018; Wang et al., 2019). We used the portal-tail complex structure segmented from the whole phage structure as the model to refine orientation and center for the portal-tail complex region in each particle image. The reconstruction and refinement were performed iteratively to improve the resolution until the orientations and centers of the portal-tail complex regions in all images were stabilized and the portal-tail complex structure could not be improved. A soft-mask was used for the region of local refinement in each particle image.

Following the same image processing protocol, we reconstructed the whole structure of the capsid II, the portal of the capsid II, and core in the mature phage and that in capsid II.

Atomic model building and refinement.

Our models of the two gp8 (in the mature phage and capsid II), gp11, gp12, and gp17 N-terminus were built based on our cryo-EM density maps using the COOT

software (Emsley et al., 2010). The models were refined using real-space refinement as implemented in Phenix (Adams et al., 2010). We built the model of gp8 in capsid II with the reference to the model of gp8 in mature phage. Refinement and validation statistics are presented in Table S1.

Supplementary text

Definition of reference orientation coordinate system of reconstruction.

The Euler angles of our portal and tail reconstruction are defined as follows. First, we place three mutually perpendicular 2-fold axes of the icosahedron coincident to the Cartesian *x*, *y*, and *z* axes, according to the icosahedral reconstruction (Baker et al., 1999; Thuman-Commike and Chiu, 2000). Then, the reference orientation coordinate system used in the symmetry-mismatch reconstruction is defined by the rotation θ=31.72° so that the axis of the tailed 5-fold vertex coincides with the z axis (Fig. S10). As such, ϕ defines the angle by which the portal-tail complex rotates around the 5-fold vertex axis of the capsid shell.

Flexible interaction between the portal and shell.

The local reconstruction method allows us to determine the orientation and center distributions of the portal with respect to the shell in the mature and capsid II particles through the use of Euler angles (Fig. S10) and 2-D translation. The Euler angles and centers (Fig. S11) show the difference between the asymmetric refinement result and the subsequent local refinement result. The rotation angles θ and ω and translations x and y of the portal position with respect to the shell in the mature particle were observed to be four narrow Gaussian distributions of particle numbers for each angles θ and ω and

translations x and y ranging from -2° to 2°and from 0 to 3 Å, respectively (Fig. S11A and S11C). These distributions can be regarded as random errors. However, the distribution of the ϕ, which represents the rotation angle of the portal around the 5-fold axis with respect to the shell, was observed to be an overlap of two Gaussian distributions with the means at -3° and 3° (a span of 6°) respectively. (Fig. S11A). It suggests that many of the particles have a wrong ϕ angle assigned to the portal (as ϕ, ϕ+72°, ϕ+144°, ϕ+216° and ϕ+288° are degenerate), requiring a change of up to 3° in either direction during local refinement.

We also determined the orientation and center distributions of the portal in the capsid II particle (Fig. S11B and S11D). All these distributions were Gaussian curves, which reflect the flexible interactions between the portal and shell and the lower resolution of this reconstruction. The ranges of the distributions of the capsid II portal were clearly wider than those of the mature phage portal. These results could not prove that the portal rotates axially, as suggested previously (Hendrix, 1978; Simpson et al., 2000).

Structural comparisons with recombinant portal and tail structures of T7.

We compared our in situ portal and tail structures with recently reported recombinant portal-tail (gp8-gp11-gp12) and portal (gp8) structures (PDB ID: 6R21, 6QXM, and 6QX5) (Cuervo et al., 2019). Our tail structure is almost identical to the reported tail structure (6R21). However, our portal in the mature phage and that in the capsid II do not fit well with any of the three portal structures. In addition, all the reported portals have no helical barrel domain.

A superposition of the recombinant portal-tail structure (6R21) with our portal-tail structure showed that the portal in the two structures are mostly identical, with the

exception of the conformation of the kinked helix (α10) and the tunnel loop. This N-terminal part of α10 in our portal structures points almost perpendicularly toward the channel axis, whereas the counterpart in their recombinant portal-tail structure tilts upward, resulting in a wider channel. In addition, the α-helices in the two crowns vary slightly. The hexameric gp12 nozzles in the two structures are identical, with the exception of a loop (residues 736-744), which was flexible in the recombinant portal-tail structure, was resolved in our structure. The gp11 proteins in the two structure have an identical main body but they have different N-terminal loops. In our structure, two adjacent gp11 N-terminal loops approach each other, resulting in a 6-fold symmetric arrangement of the gp11 dimer (Fig. S6B and S6C). These structural variations occur in the regions interacting with the gp17 trimers, which were absent from their structures. The portal structure 6QX5 is similar to our portal structure in the mature phage, although most of the secondary structure does not fit well. The portal structure 6QXM is similar to our portal structure in the capsid II, however, the crown is not present in their portal, and the N-terminal part of α10 in their portal structure tilts upward (in contrast to the rough perpendicularity toward the channel axis in our structure).

Legends for Movies S1.

Conformational change of portal from packaging state to mature state.

SI References:

Adams, P.D., Afonine, P.V., Bunkoczi, G., Chen, V.B., Davis, I.W., Echols, N., Headd, J.J., Hung, L.W., Kapral, G.J., Grosse-Kunstleve, R.W., *et al.* (2010). PHENIX: a comprehensive Python-based system for

macromolecular structure solution. Acta Crystallogr D Biol Crystallogr 66, 213-221.
Baker, T.S., Olson, N.H., and Fuller, S.D. (1999). Adding the third dimension to virus life cycles: three-dimensional reconstruction of icosahedral viruses from cryo-electron micrographs. Microbiol Mol Biol Rev 63, 862-922.
Cuervo, A., Fabrega-Ferrer, M., Machon, C., Conesa, J.J., Fernandez, F.J., Perez-Luque, R., Perez-Ruiz, M., Pous, J., Vega, M.C., Carrascosa, J.L., *et al.* (2019). Structures of T7 bacteriophage portal and tail suggest a viral DNA retention and ejection mechanism. Nat Commun 10, 3746.
Emsley, P., Lohkamp, B., Scott, W.G., and Cowtan, K. (2010). Features and development of Coot. Acta Crystallogr D Biol Crystallogr 66, 486-501.
Fuller, S.D., Butcher, S.J., Cheng, R.H., and Baker, T.S. (1996). Three-dimensional reconstruction of icosahedral particles - The uncommon line. Journal of Structural Biology 116, 48-55.
Hendrix, R.W. (1978). Symmetry mismatch and DNA packaging in large bacteriophages. Proc Natl Acad Sci U S A 75, 4779-4783.
Kivioja, T., Ravantti, J., Verkhovsky, A., Ukkonen, E., and Bamford, D. (2000). Local average intensity-based method for identifying spherical particles in electron micrographs. J Struct Biol 131, 126-134.
Li, X., Mooney, P., Zheng, S., Booth, C.R., Braunfeld, M.B., Gubbens, S., Agard, D.A., and Cheng, Y. (2013). Electron counting and beam-induced motion correction enable near-atomic-resolution single-particle cryo-EM. Nat Methods 10, 584-590.
Li, X., Zhou, N., Chen, W., Zhu, B., Wang, X., Xu, B., Wang, J., Liu, H., and Cheng, L. (2017). Near-atomic resolution structure determination of a cypovirus capsid and polymerase complex using cryo-EM at 200kV. Journal of Molecular Biology 429, 79-87.
Liu, H., and Cheng, L. (2015). Cryo-EM shows the polymerase structures and a nonspooled genome within a dsRNA virus. Science 349, 1347-1350.
Morais, M.C., Tao, Y., Olson, N.H., Grimes, S., Jardine, P.J., Anderson, D.L., Baker, T.S., and Rossmann, M.G. (2001). Cryoelectron-microscopy image reconstruction of symmetry mismatches in bacteriophage phi29. J Struct Biol 135, 38-46.
Simpson, A.A., Tao, Y., Leiman, P.G., Badasso, M.O., He, Y., Jardine, P.J., Olson, N.H., Morais, M.C., Grimes, S., Anderson, D.L., *et al.* (2000). Structure of the bacteriophage phi29 DNA packaging motor. Nature 408, 745-750.
Thuman-Commike, P.A., and Chiu, W. (1997). Improved common line-based icosahedral particle image orientation estimation algorithms. Ultramicroscopy 68, 231-255.
Thuman-Commike, P.A., and Chiu, W. (2000). Reconstruction principles of icosahedral virus structure determination using electron cryomicroscopy. Micron 31, 687-711.
Wang, J., Yuan, S., Zhu, D., Tang, H., Wang, N., Chen, W., Gao, Q., Li, Y., Wang, J., Liu, H., *et al.* (2018). Structure of the herpes simplex virus type 2 C-capsid with capsid-vertex-specific component. Nat Commun 9, 3668.
Wang, N., Zhao, D., Wang, J., Zhang, Y., Wang, M., Gao, Y., Li, F., Wang, J., Bu, Z., Rao, Z., *et al.* (2019). Architecture of African swine fever virus and implications for viral assembly. Science 366, 640-644.
Yuan, S., Wang, J., Zhu, D., Wang, N., Gao, Q., Chen, W., Tang, H., Wang, J., Zhang, X., Liu, H., *et al.* (2018). Cryo-EM structure of a herpesvirus capsid at 3.1 A. Science 360.

Zhu, D., Wang, X., Fang, Q., Van Etten, J.L., Rossmann, M.G., Rao, Z., and Zhang, X. (2018). Pushing the resolution limit by correcting the Ewald sphere effect in single-particle Cryo-EM reconstructions. Nat Commun 9, 1552.

Supplementary Figures

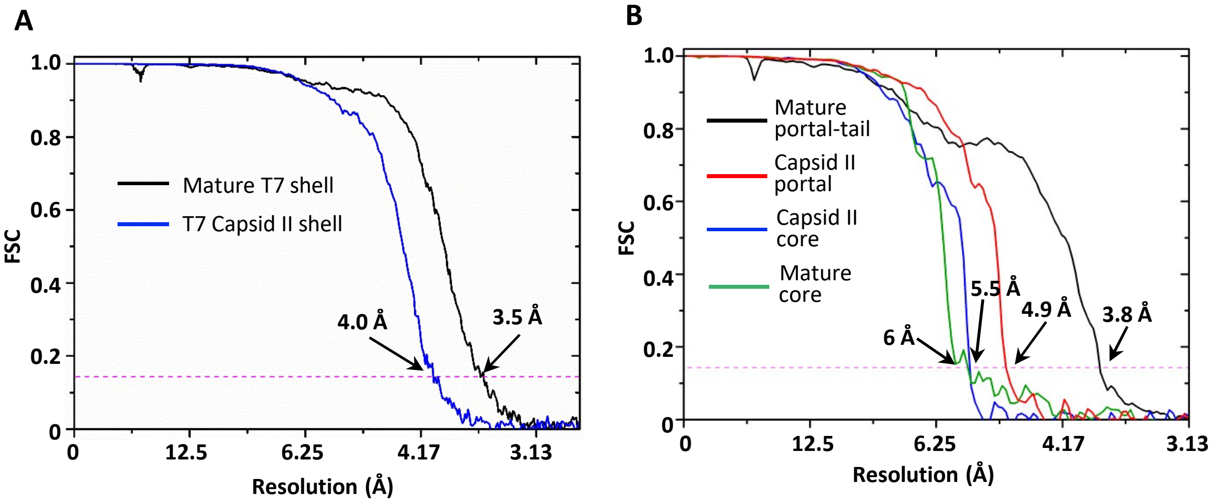

Fig. S1. Fourier shell correlation (FSC) curves. We randomly split the whole dataset in two halves for calculating the FSC. Soft-masks were used for the resolution assessments. (A) Estimated structural resolutions of the icosahedral capsid shells of the mature T7 and capsid II. (B) The estimated structural resolutions of the core, portals, and tail in the mature T7 and capsid II.

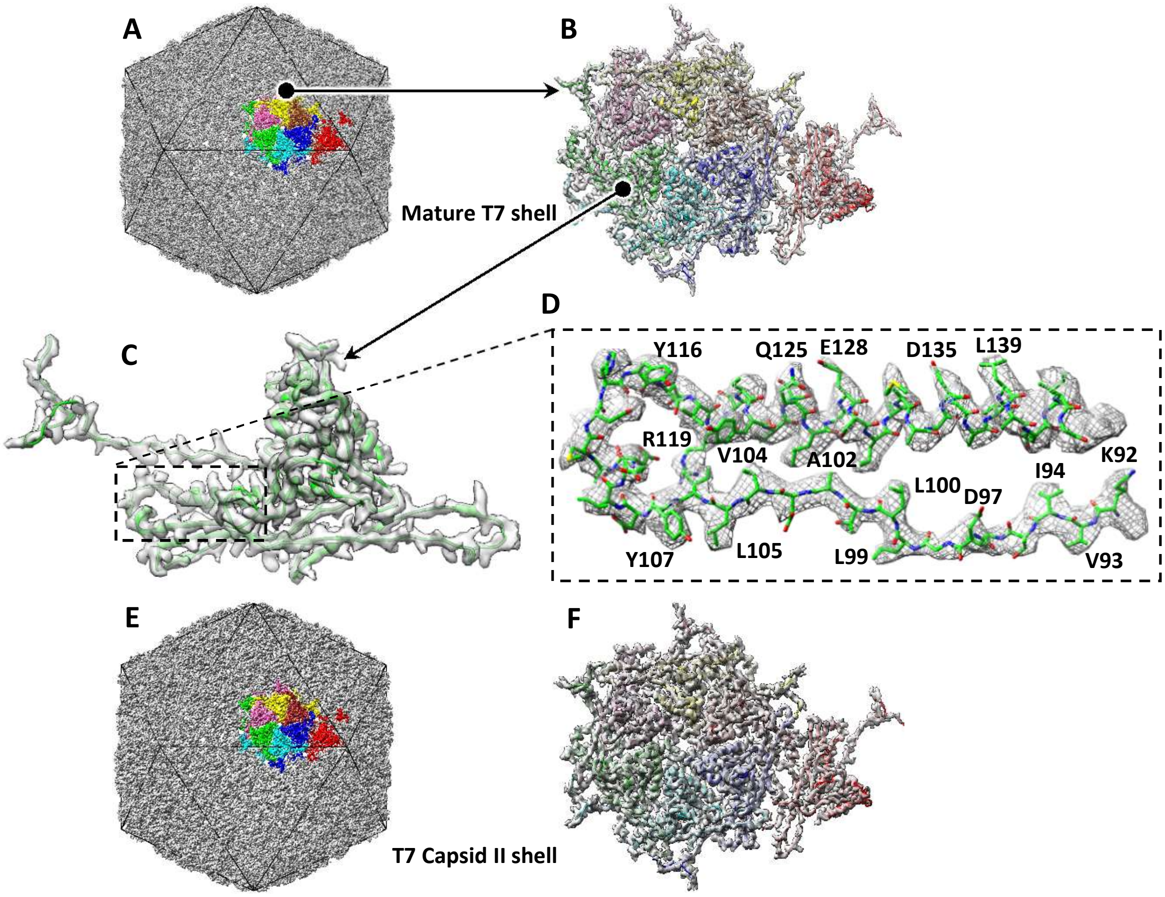

Fig. S2. Icosahedral reconstruction of the mature T7 and capsid II. (A) Overall view of the mature T7 icosahedral capsid shell structure. Capsid shell proteins in an asymmetric unit are colored. (B) Zoomed-in view of the asymmetric unit (transparent view) superimposed with atomic model of the capsid shell proteins (ribbon view). (C) Zoomed-in view of a shell protein (transparent view) superimposed with its atomic model (ribbon view). (D) Zoomed-in view of a protein segment (mesh view) superimposed with its atomic model (stick view). (E) Overall view of the icosahedral capsid II shell structure. (F) Zoomed-in view of the asymmetric unit (transparent view) superimposed with atomic model of the capsid shell proteins (ribbon view).

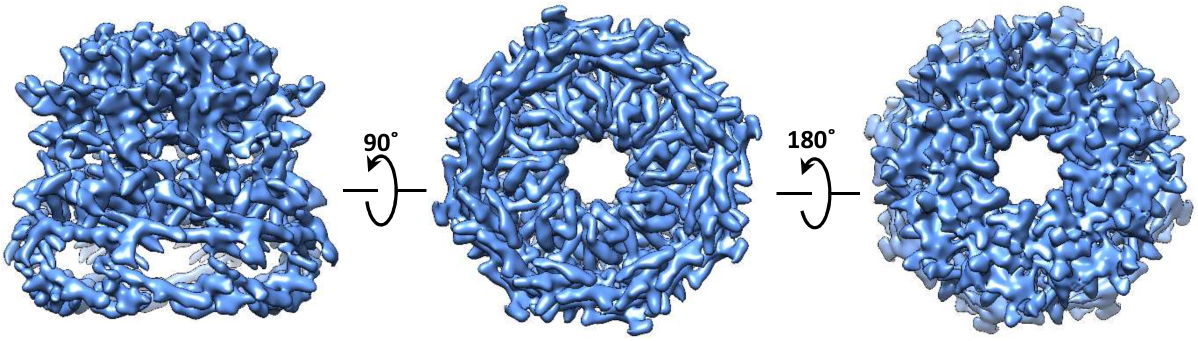

Fig. S3 Side, bottom, and top views of the 8-fold core in the mature phage show that the bottom region is a helix-rich structure.

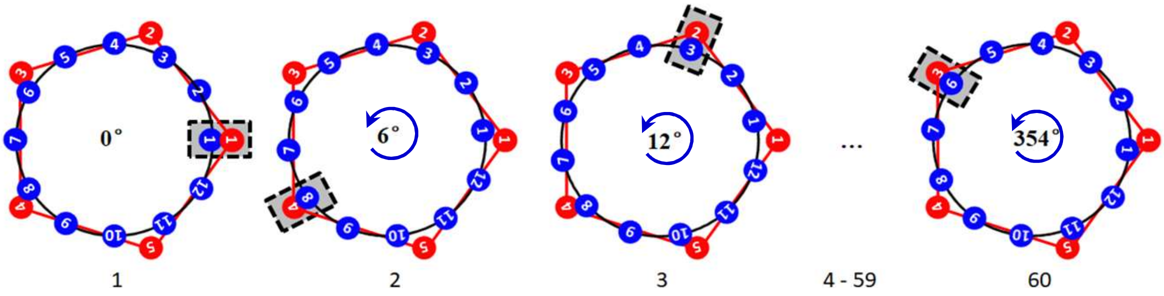

Fig. S4. A diagram of the symmetry-mismatch interaction between the 5-fold shell and 12-fold portal. When the portal (blue) rotates 6 degrees, the portal-shell interaction will coincide with the portal-shell interaction before the rotation.

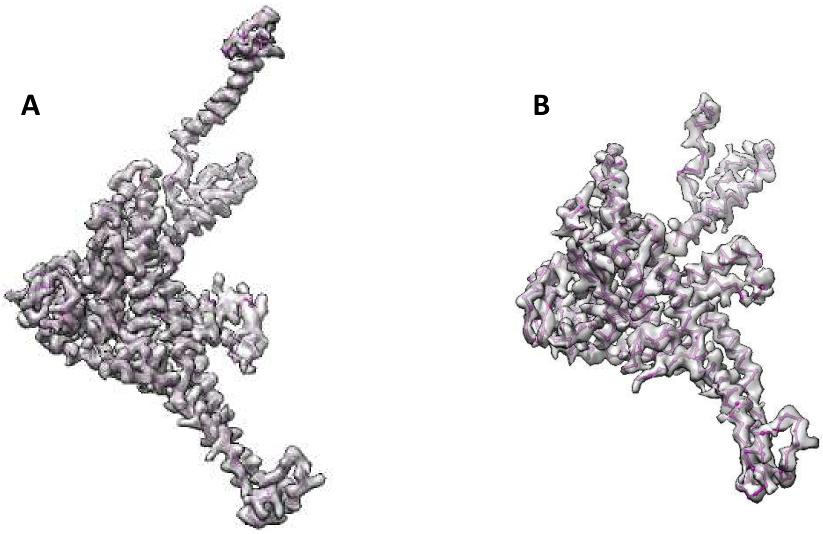

Fig. S5. Superposition of a copy of the portal (gray) in mature T7 phage (A) and in capsid II (B) on its atomic model (magenta) .

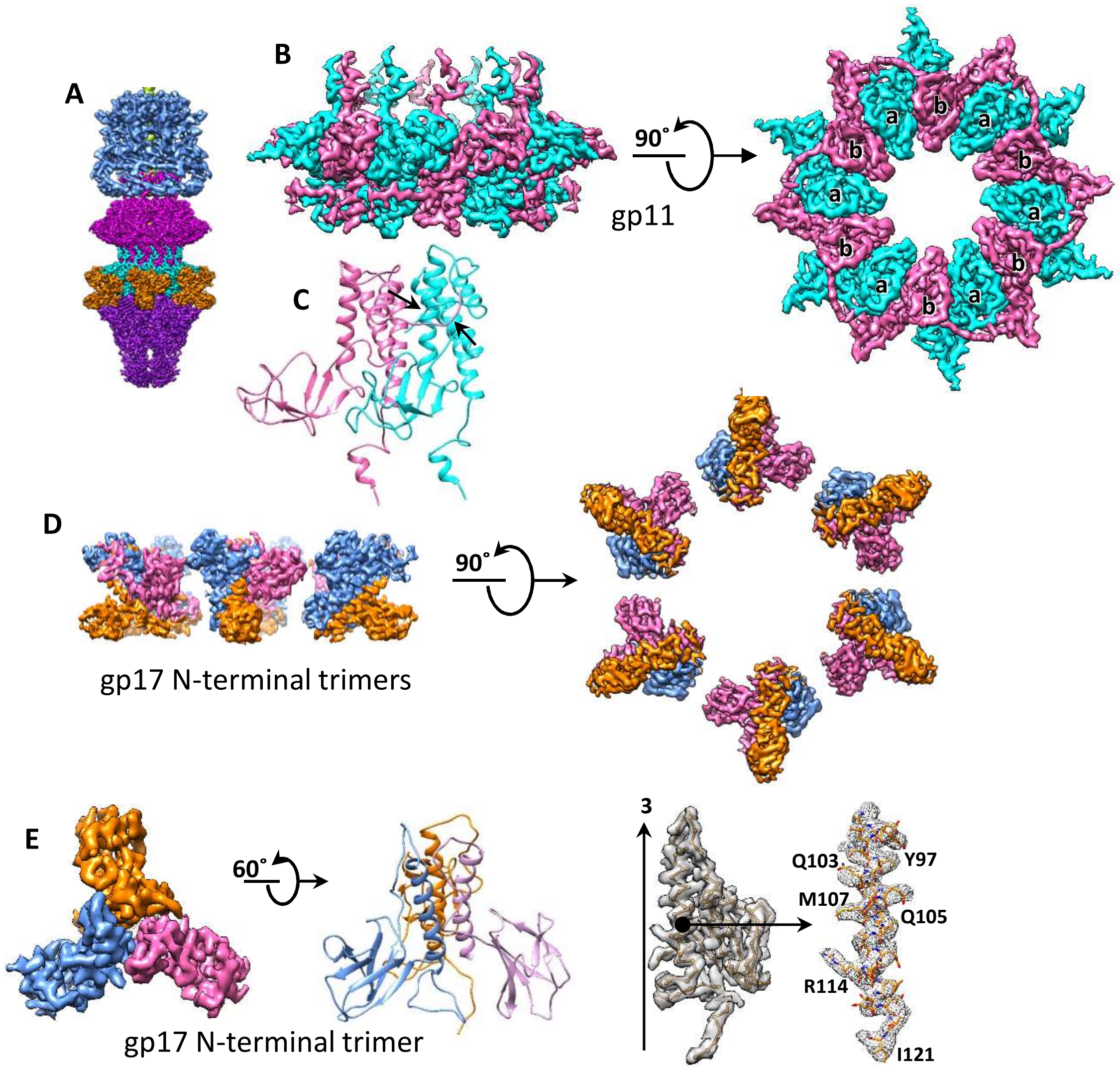

Fig. S6. Structures of the tail complex. (A) Overall view of the core, portal, and tail complex in the mature phage. (B) 12-fold gp11 proteins. (C) Two adjacent gp11 N-terminal loops approach each other (indicated by two arrows in the ribbon view). (D) 6-fold gp17 N-terminal trimmers. (E) Zoomed-in view of the gp17 trimer and monomer.

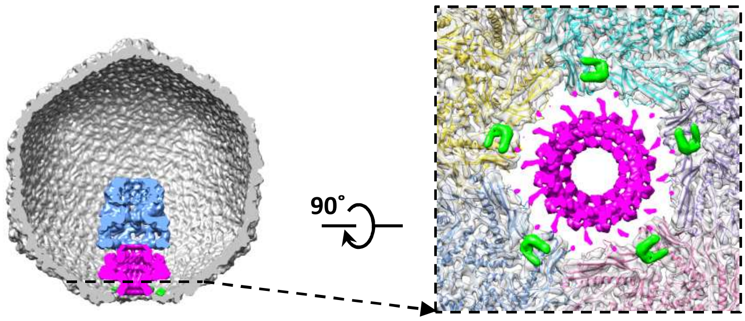

Fig. S7 Zoomed-in view of the portal-shell interaction. Five copies of the two α-helices are in green. Fitting of shell proteins (colored ribbons) into the density map (gray transparent view) reveals that the helices cannot be assigned to the shell proteins. The helices could be assigned to part of the scaffolding protein.

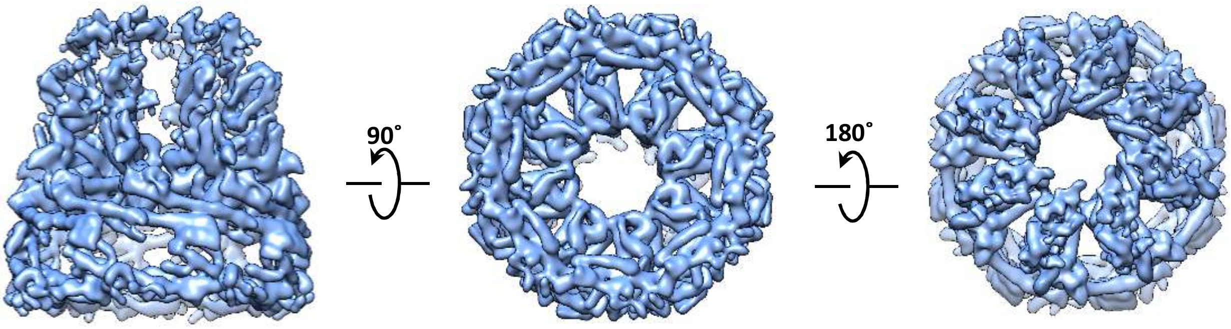

Fig. S8 Side, bottom, and top views of the core structure in the capsid II.

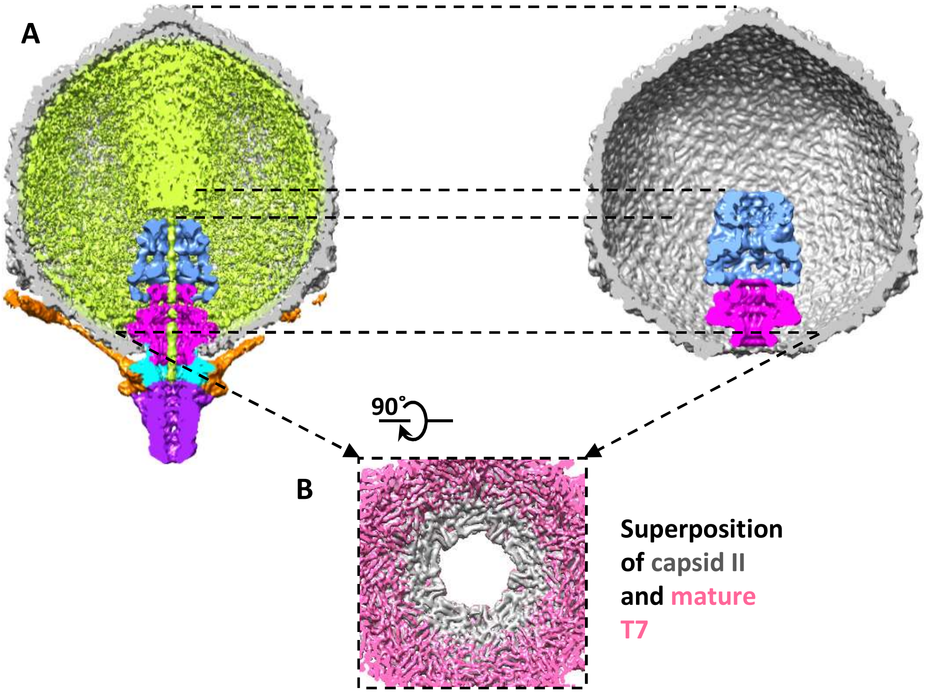

Fig. S9. Conformational changes between mature phage and capsid II. (A) Cut-open views of the mature phage (left) and the capsid II (right) show that the portal and core in mature phage move downward. (B) Cut-open view of superposition of the capsid II (gray) and mature phage (pink) shows that the open vertex of the shell is squeezed out by the portal during maturation. The open vertex structure was improved to 8 Å resolution by using the local reconstruction. The portal structures were not shown for clarity.

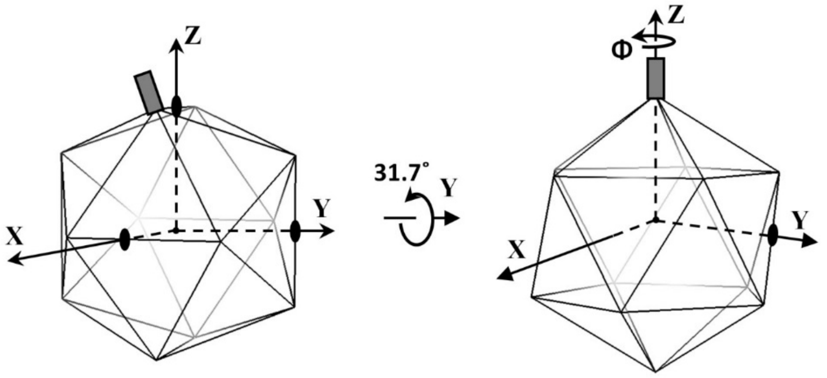

Fig. S10. Definitions of reference orientation coordinate systems for icosahedral (left) and asymmetrical (right) reconstructions.

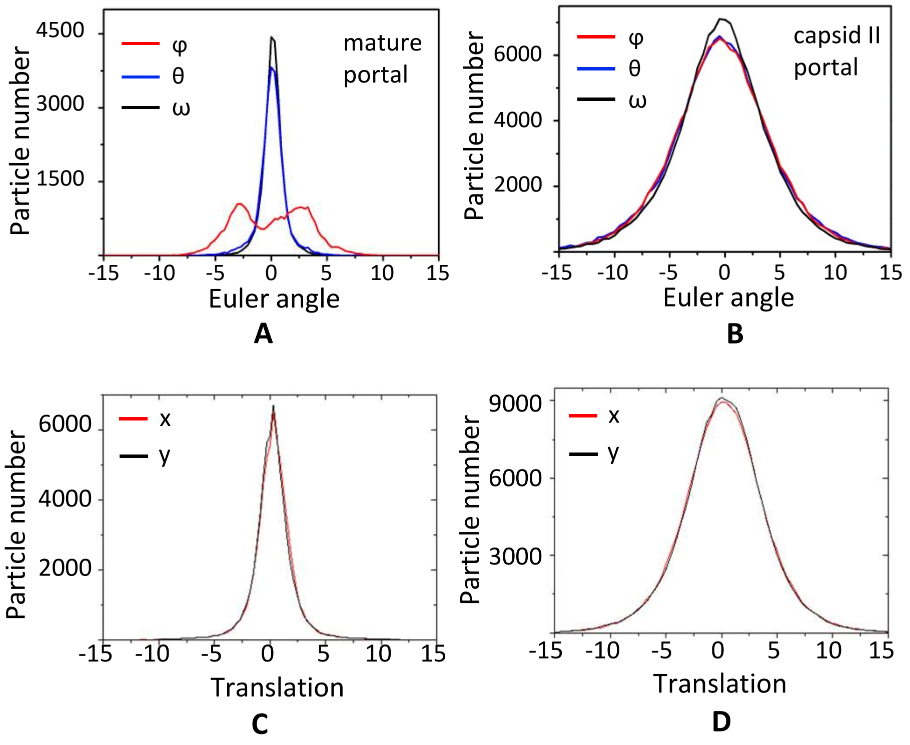

Fig. S11. Orientation and center distributions of portal in the mature and capsid II particles. For panels A and B, the vertical axis represents the number of particles, and the horizontal axis represents three Euler angles of the portal rotation in degree. Only the period of φ (360/12=30° for the 12-fold portal) was plotted. For panels C and D the vertical axis represents the number of particles, and the horizontal axis represents the portal translations in pixel (pixel size is 1.27 Å). (A) Distributions of the portal orientation angles with respect to the shell in mature particle. (B) Distributions of the portal orientation angles with respect to the shell in capsid II particle. (C) Distribution of the portal translations with respect to the shell in the mature particle. (D) Distribution of the portal translations with respect to the shell in the capsid II particle.

Table S1. Data collection, reconstruction, and modeling statistic.

**Data collection**						
Electron microscopy	FEI 200 kV Technai Arctica, Falcon II camera					
Pixel size	1.27Å					
Defocus range	0.5 to 4.0um					
Total movie-mode micrographs	mature phage T7				capsid II	
	1,785				5,175	
**Icosahedral reconstruction**						
	mature phage T7				capsid II	
Total particles	58,690				84,318	
Resolution	3.5Å				4.0Å	
B-factors	200				240	
EMDB ID	EMD-30139				EMD-30142	
Symmetry-mismatch reconstruction						
	mature phage T7				capsid II	
Total particles	57,954				71,564	
Resolution	7				10	
EMDB ID	EMD-30140				EMD-30144	
Local reconstruction						
	mature phage T7				capsid II	
	portal-tail			core	portal	core
Total particles	55,212			44,870	84318	69,964
Resolution	3.8Å			6Å	4.9Å	5.9Å
B-factors	160			0	180	0
EMDB ID	EMD-30134,30135,30136,30137			EMD-30141	EMD-30138	EMD-30145
Atomic models refinement/statistics (phenix.real_space_refine）						
	mature phage T7				capsid II	
Protein	gp8	gp11	gp12	gp17	gp8	
PDB ID	7BOU	7BOX	7BOY	7BOZ	7BP0	
Model Resolution in Refinement	4.0 Å	4.0 Å	4.0 Å	4.0 Å	5.0 Å	
Total Residues	6252	2310	4734	2268	5136	
CC (model to map fit)	0.8096	0.8041	0.8201	0.7907	0.7159	
Ramachandran most favorable (%)	92.03	92.73	85.33	91.05	81.71	
Ramachandran additionally allowed (%)	5.27	6.49	10.94	6.31	14.49	
Ramachandran disallowed (%)	2.7	0.78	3.73	2.64	3.8	
